# Supplementary material for: Synthesis of TiO2/WO3 Composite Nanofibers by a Water-Based Electrospinning Process and Their Application in Photocatalysis
Source: Nanomaterials (Basel). 2020 May 2;10(5):882. doi: 10.3390/nano10050882 (PMC7279266; doi:10.3390/nano10050882)
Supplement: Supplementary file 1 [file nanomaterials-10-00882-s001.pdf]

Supplementary Materials:

Synthesis of TiO_2/WO_3 Composite Nanofibers by a Water-Based Electrospinning Process and Their Application in Photocatalysis

Vincent Otieno Odhiambo [1,*], Aizat Ongarbayeva [1], Orsolya Kéri [1], László Simon [2] and Imre Miklós Szilágyi [1]

[1] Department of Inorganic and Analytical Chemistry, Budapest University of Technology and Economics, H-1111 Budapest, Szent Gellért tér 4., Hungary; ayzatonga@gmail.com (A.O.); orsolyakeri@gmail.com (O.K.); imre.szilagyi@mail.bme.hu (I.M.S.)

[2] Department of Organic Chemistry and Technology, Budapest University of Technology and Economics, H-1111 Budapest, Budafoki út 8., Hungary; simon.laszlo92@gmail.com

* Correspondence: vincent.odhiambo@mail.bme.hu

Percentage of semi-conductor oxides in the precursor

TiO_2

$$C_6H_{18}N_2O_8Ti \rightarrow TiO_2 + N_xO_y + CO_{x-1} + nH_2O$$

$$\frac{79.87}{294.08} \times 100 = 27.16\%$$

WO_3

$$(NH_4)6[H_2W_{12}O_{40}]xH_2O \rightarrow 12WO_3 + 6NH_3 + (x+4)H_2O$$

$$\frac{2781.6}{2956.30} \times 100 = 94.08\%$$

Tauc plots

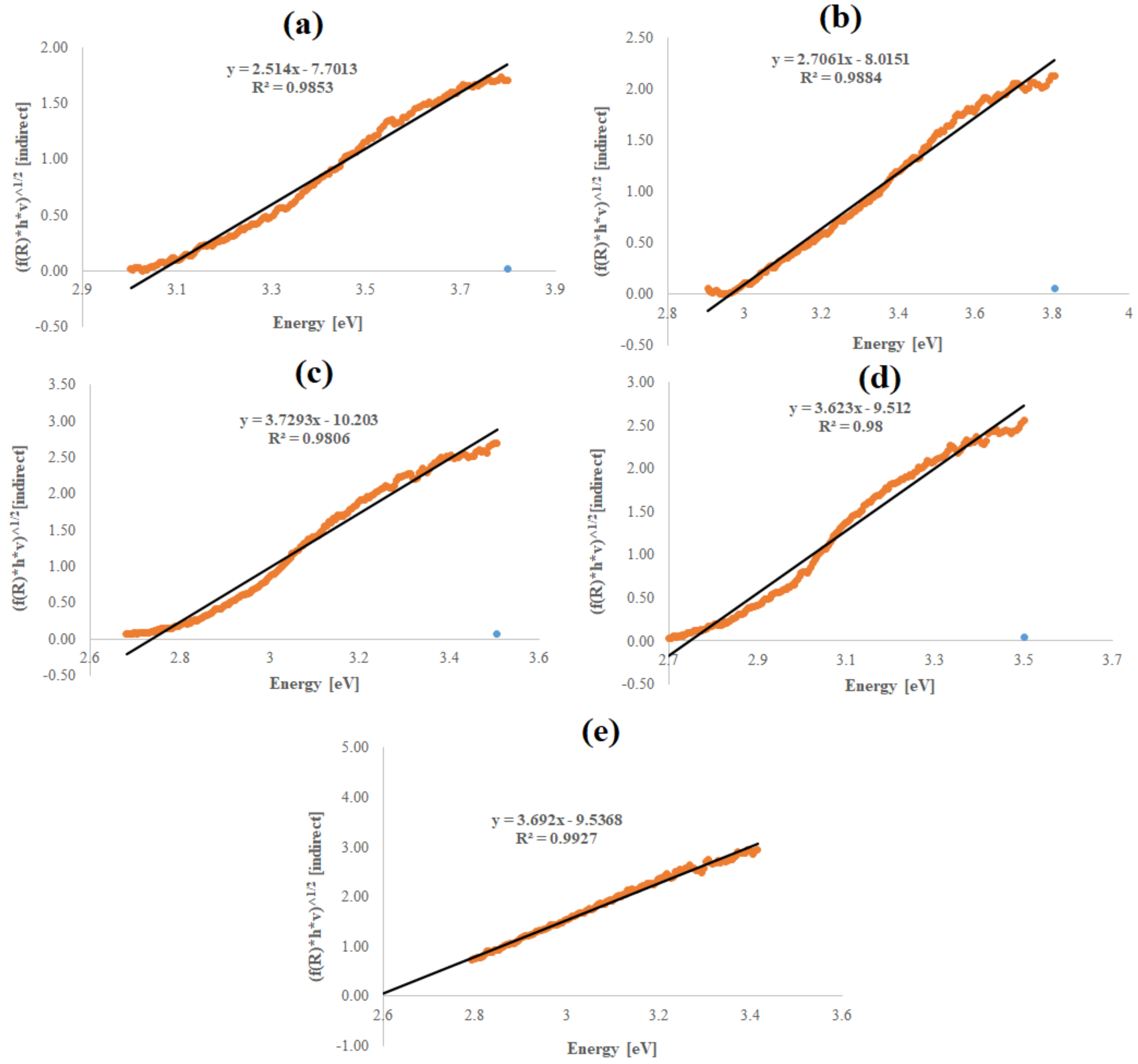

Figure 1. Tauc plots for annealed fibers (**a**) 100% TiBALDH (**b**) 90% TiBALDH (**c**) 50% TiBALDH (**d**) 10% TiBALDH (**e**) 0% TiBALDH.

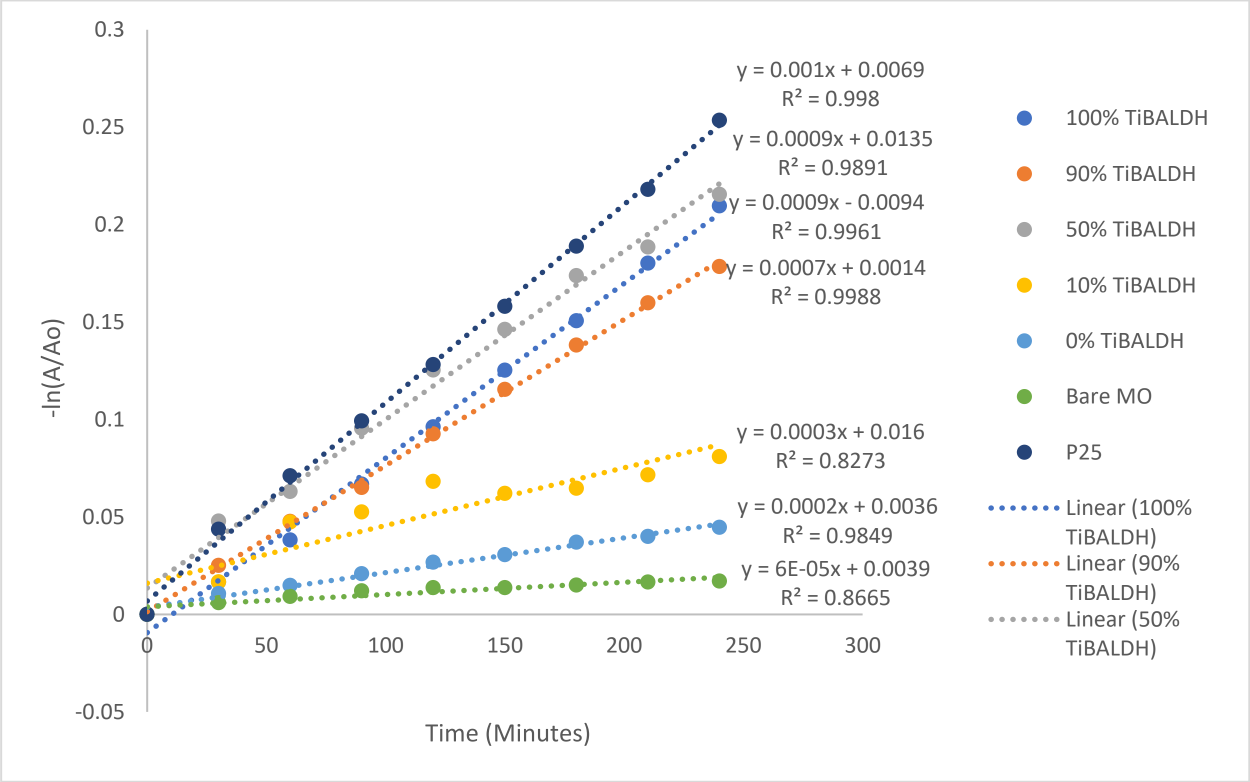

Figure 2. Apparent rate constant(slope) and r² values for the photocatalytic degradation of methyl orange in UV light.

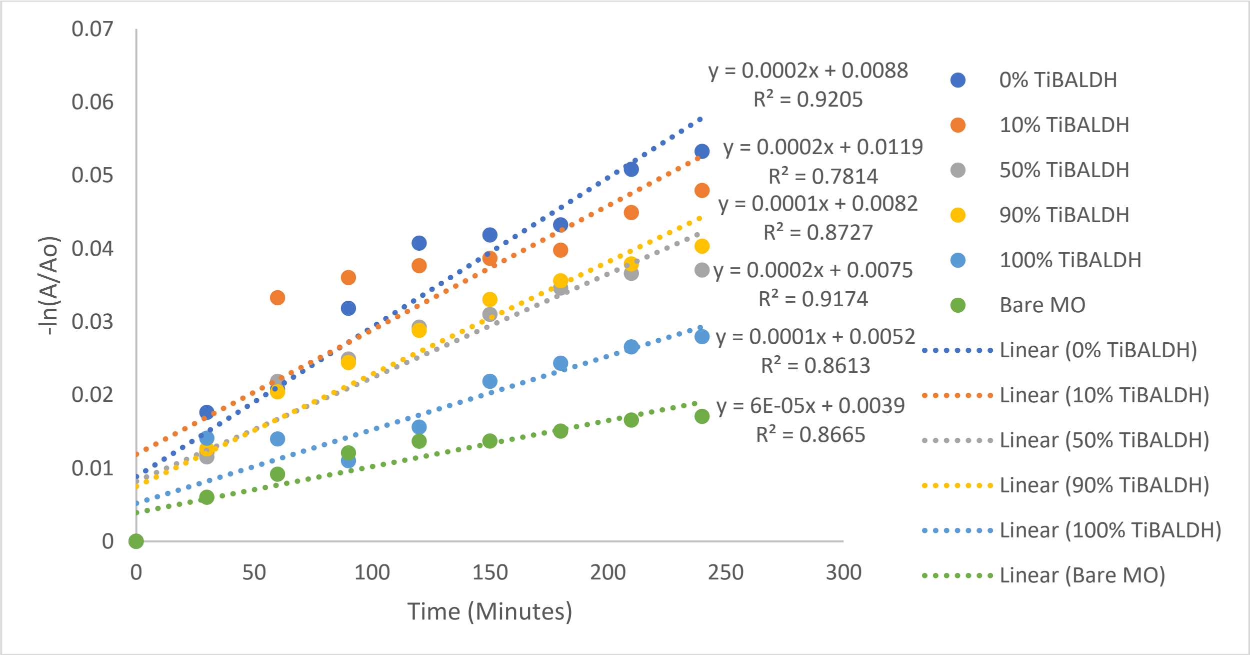

Figure 3. Apparent rate constant(slope) and r² values for the photocatalytic degradation of methyl orange in Visible light.
